# The Role of Preharvest Natural Infection and Toxin Contamination in Food and Feed Safety in Maize, South-East Hungary, 2014–2021

**DOI:** 10.3390/jof8101104

**Published:** 2022-10-19

**Authors:** Akos Mesterhazy, Denes Szieberth, Eva Toldine Tóth, Zoltan Nagy, Balazs Szabó, Beata Herczig, Istvan Bors, Beata Tóth

**Affiliations:** 1 Cereal Research Non-Profit Ltd., Fesu Street 1, 6701 Szeged, Hungary; 2 Hungarian Maize Club, Kazinczy Str. 12, 8152 Kőszárhegy, Hungary; 3 Bonafarm-Babolna Feed Ltd., Laboratory Branch, 2942 Nagyigmand, Hungary

**Keywords:** preharvest mycotoxins, *Fusarium graminearum*, *Fusarium verticillioides*, *Aspergillus flavus*, deoxynivalenol, fumonisin, aflatoxin, natural infection, natural toxin contamination, changing environmental conditions

## Abstract

Mycotoxins originating in the preharvest period represent a less studied research problem, even though they are of the utmost practical significance in maize production, determining marketability (within EU limits), and storage ability, competitiveness, and profit rate. In this study, 18–23 commercial hybrids were tested between 2014 and 2021. Natural infection from *Fusarium* spp. was higher than 1.5%, and for *Aspergillus* spp. this was normally 0.01% or 0, much lower than would be considered as severe infection. In spite of this, many hybrids provided far higher toxin contamination than regulations allow. The maximum preharvest aflatoxin B_1_ was in 2020 (at 2286 μg/kg), and, in several cases, the value was higher than 1000 μg/kg. The hybrid differences were large. In Hungary, the presence of field-originated aflatoxin B_1_ was continuous, with three AFB1 epidemics in the 8 years. The highest DON contamination was in 2014 (at 27 mg/kg), and a detectable DON level was found in every hybrid. FUMB_1_+B_2_ were the highest in 2014 (at 45.78 mg/kg). At these low infection levels, correlations between visual symptoms and toxin contaminations were mostly non-significant, so it is not feasible to draw a conclusion about toxin contamination from ear rot coverage alone. The toxin contamination of hybrids for a percentage of visual infection is highly variable, and only toxin data can decide about food safety. Hybrids with no visual symptoms and high AFB1 contamination were also identified. Preharvest control, including breeding and variety registration, is therefore of the utmost importance to all three pathogens. Even natural ear rot and toxin data do not prove differences in resistance, so a high ear rot or toxin contamination level should be considered as a risk factor for hybrids. The toxin control of freshly harvested grain is vital for separating healthy and contaminated lots. In addition, proper growing and storage conditions must be ensured to protect the feed safety of the grain.

## 1. Introduction

Toxigenic fungi cause severe economic losses during maize production, and the preharvest or postharvest occurrence of all toxin limits must be measured. When in the field, the preharvest character dominates breeding in terms of resistance, fungicide protection, etc., and provides valuable information toward their control. As in Hungary *A. flavus* is a new problem of possible preharvest origin, it was the focus of this paper. However, near to no published data are known about the preharvest presence of DON and fumonisins, and they occur regularly, as even farmers know about this problem, and therefore, these toxins have to receive the same attention. This is the reason that all three toxins are controlled, and so epidemiological data of their significance can also be presented.

Christensen and Kaufmann [1] divided fungi into two categories: fungi isolated from grains for storage and field fungi. Species from *Alternaria*, *Cladosporium*, *Helminthosporium*, and *Fusarium* spp. Are classified as field fungi. The EU regulations [2,3,4] indicate the binding limits for food and suggest limits for feeds. As these limits are different for humans and also differ between animals, we used the limits for swine as they are the most sensitive to toxins (suggested EU feed limits for adults: DON 0.9 mg/kg, FUM 5 mg/kg, and aflatoxin 20 μg/kg; As swines are as sensitive to toxins as human beings, for piglets, about 20–25% of the limit defined for adult swine would be sufficient. The regulations do not differentiate between preharvest and postharvest contamination. However, in this paper, we test the preharvest origin as this will help to identify the field originated risks and this is the first point from where field-originated toxin problems can be identified and the best solution to reduce this problem can be found.

Among field-borne toxigenic fungi, *Fusarium* species are well-known toxin producers [1,5,6]. Past research has also shown that their toxins are also field-borne, so no additional proof is provided [1,5,7]. The proposition that field-borne infection decreases during storage could be true, but this is not necessarily right for all mycotoxins [5,6].

In Hungary, artificially inoculated wheat spikes of *F. graminearum* showed a concentration as high as 432 mg/kg of DON, but zearalenone was never found [8]. Munkvold and White [7] noted that aflatoxins have both field and storage significance. Moreover, aflatoxin production in the field in the USA is so important that *A. flavus* was classified among the field ear rots [7]. For this reason, we treat *A. flavus* in the preharvest group and included into the resistance studies. However, for preharvest aflatoxin occurrence, no reliable data have been published from Hungary, and thus this problem needed a solution.

Prior to 2004, the European literature did not recognize aflatoxins in the Mediterranean area. The U.S. literature has reported [4] high aflatoxin contamination in the USA under southern tropical and subtropical climatic conditions. Shotwell [9] reported that, in 1964 and 1965, 2.3%of 1311 corn samples were contaminated by aflatoxins. The samples were commodities, and the preharvest origin of the aflatoxin was not demonstrated. The first evidence of aflatoxins in preharvest maize was published in 1975. Anderson et al. [10] reported aflatoxin levels exceeding 400 mg/kg in individual kernels under artificial inoculation, thereby demonstrating the possibility that aflatoxins can be produced under field conditions. Lillehoj et al. [11] were the first to collect 3600 ears of maize from fields and found that 120 had aflatoxin levels higher than 20 μg/kg. Subsequently, aflatoxin has also been considered a field-borne mycotoxin in the USA [12,13,14]. Lillehoj [15] was among the first to report differences in aflatoxins in various maize hybrids.

Abbas et al. [16] performed tests following natural infection from fumonisins and aflatoxin, a rare case for evaluating visual symptoms, but the relation between symptoms and toxin contamination was not analyzed. In some experiments, a positive correlation was found between aflatoxin and fumonisin contamination, but the correlated value was only r=0.298, with no statistical significance. Further, Abbas et al. [16] concluded that the same cultural practices may influence differently aflatoxin and fumonisin contamination. In Turkey [17], 19.3% of the isolates from freshly harvested maize grains belonged to *Aspergillus flavus*. In another study, aflatoxins were detected in 17 out of the 73 samples collected (0.7–50 μg/kg). In another test, 46% of the samples contained 3–70 μg/kg of aflatoxins [17], but visual notes were not reported. Between 2000 and 2003, Abbas et al. [18] found significantly lower natural aflatoxin levels (1 to 2 μg/kg), with the maximum being 9.2 μg/kg. Abbas et al. [19] found that the common smut infection by *Ustilago maydis* can increase aflatoxin contamination by 45-fold, whereas the concentration of fumonisins increased only 5.2-fold. Lillehoj [20] reported that earlier studies concentrated on stored commodities, as *A. flavus* and *A. parasiticus* were classified as storage fungi. The discovery of preharvest infection and aflatoxin contamination in the field opened a new avenue of research in mycotoxicology. This shift caused a radical reorientation in scientific thinking. The consequence was the research on aflatoxin production and its conditions [21,22].

The preharvest origin of the aflatoxin has not been considered a central problem [23]. In Hungary [24], until 2006, no aflatoxins were reported in maize. Masic et al. [25] reported on maize samples from Hungary, but the status of the samples (preharvest or stored) was not provided. In Hungary, data on 17,011 maize samples regarding mycotoxins was published from 2012 to 2017 [26]. Aflatoxins were found every year, with maximums between 0.44 and 115 μg/kg. As the data originated mostly from stored mixed corn samples, no conclusions could be drawn about the preharvest or postharvest origins of the contamination or the possible role of resistance [26]. Therefore, aflatoxin was included into the tested toxins. When preharvest occurrence is continuous and significant, breeding, agronomy, etc. may have a role in its control.

In areas where aflatoxin was demonstrated to have a field origin, such as in the southeastern parts of the USA and tropical regions, the search for sources of resistance started decades before [15,20,27,28,29]. In these areas, Munkvold and White [7] maintain that preharvest aflatoxin contamination is more important than storage-borne contamination. Unsurprisingly, nearly everywhere, following artificial inoculation, aflatoxin contamination has been accompanied by studies on the development of the disease resistance. However, from these cannot help to demonstrate the preharvest natural contamination and its significance.

The ecological conditions supporting the toxigenic diseases and the regulation of the toxin contamination are roughly known [7,30,31,32,33,34]. The data provide the variety differences to the diseases, but there is no general knowledge in this field. The existing risk is sufficiently enough to receive attention, but increasing temperatures in west and north Europe will cause increasing DON contamination [35], and higher fumonisin and aflatoxin contamination is also forecast. Monitoring is important in order to detect the problem before it spreads to the more jeopardized regions.

We should not forget that the forecasting refers to the preharvest toxin contamination. As *A. flavus* and *F. verticillioides* need a higher optimum temperature than the ruling weather conditions secured in middle and western Europe, warmer and dryer summer conditions will increase the risk of aflatoxin and fumonisin contamination [36,37]. The different forecasting models [35,38,39,40,41,42] do not consider the resistance of the hybrids but use only the increasing temperatures and other non-plant traits. Accordingly, significant aflatoxin contamination is forecast in middle, west, and towards northern European regions. However, on the other hand, a number of papers provided adequate proof on the differences in resistance for each important toxigenic fungus [43]. Other authors [29,30,44,45,46] have recognized that there is higher toxin contamination in warmer and drier years. However the possibility of the higher resistance has not been considered as a possible control method. Rather, authors think that biocontrol using an atoxic *A. flavus* strain [46,47] can be more successful. In Hungary, Mesterhazy et al. (2022) [48] published artificial inoculation result for *F. graminearum, F. verticillioides*, and *A. flavus.* In the non-inoculated sample, DON, FUM B_1_+B_2,_ and AFB1 were also controlled. For the natural toxin contamination, only the mean values were presented (2017–2020), but for the entire period (2014-2021), the yearly data are important to gain a better understanding of the nature of the natural toxin contamination. The conclusion is that the resistance level or contamination level of the hybrids should be considered to see how far a forecasting can be valid for all hybrids, and how relevant it is to forecast toxin contamination without resistance data. The work in [41] is important because it showed that beside the temperature also humidity is needed for disease spread and toxin contamination. The toxin contamination for a percentage of the visual scores was recognized in artificial inoculation tests [48]; in this paper, we test this for natural infection.

Storage is a key problem. In Hungary, regularly stored and moldy corn samples were compared in 1993–1999 [24]. The data are important (Table 1), because the assumption that field-originated toxins do not increase during storage [1] seems to be false. In a bad storage environment, there is, on average, a two- to eight-fold increase in the levels of different mycotoxins. During regular storage, a lower level of mycotoxin contamination increase was observed. In bad storage conditions, a sharp increase in toxins was documented. In other words, it appears storability is better when the starting mycotoxin conditions of the grain are healthier. Unfavorable storage conditions can considerably increase the mycotoxin contamination caused by field fungi.

Objectives. The main task of this study is to monitor the significance of the natural preharvest contamination of mycotoxins, including aflatoxin, DON, and fumonisins, in South Hungary. The assessment of the differences among possible hybrids was also conducted. The relations between infection severity and toxin contamination are mostly unknown at this low infection level. The data can also contribute to the epidemiological knowledge of the three toxins. We also considered the possibilities of identifying hybrids that adapt better to different ecological situations, and we expect the study to contribute to a more extended use of preharvest control methods. Similarly, we expect to see an increased role of preharvest contamination in the plant production process.

## 2. Materials and Methods

For long, natural infections have been the basis for selecting plants with higher resistance. However, it has become clear [43] that natural infections and toxin contamination are not suitable bases to conclude that there are resistance differences, since for natural infection, the inoculation time is unknown and meteorological conditions change through the 2–3 weeks of the flowering period. However, the explicit role of toxin contamination and the rather complicated interactions in the trade, food, and feed industry need to be better understood to be able to find ways improving food and feed safety in maize. This was the reason why we took toxin measurements and evaluated visual notes. We could not draw any definite conclusions about resistance to disease and toxins; thus, we do not exclude them as a possible explanation for the hybrid differences. Rather, we looked for new ways to use these data in maize production and technology.

### 2.1. Field Tests

#### 2.1.1. Plant Material

Yearly, 18–23 international commercial maize hybrids are tested. These are also registered in Hungary. No resistance data are obtained from the hybrids. This was why we conducted this work. Our intention was to identify hybrids that have a low to medium risk of mycotoxin contamination. Initially, the number of overlapping hybrids was 5–6. In 2017/2018 and 2019/2020, 18 hybrids were identical, and 4 control hybrids were used over the four years. The hybrids could not be provided with a resistance designation, as public data do not exist (except in rare cases).

The experimental samples were sown at the Kiszombor experimental station in the Maros river valley, 25 km east of Szeged Cereal Research, Ltd., Szeged, Hungary (GPS coordinates: 46°12′49.0′′ N, 20°09′57.9′′ E). The clay content was high, the humus content was 3%, and the yearly precipitation was 600 mm/year, varying between 350 and 1100 mm. In all, 160 kg of complex fertilizer (Genesis) was added to 8% and 21% nitrogen, phosphorus, and potassium in the autumn and 80 kg a.i. (Nitrosol, 46% carbamide) in the spring. Irrigation was carried out in spring when it was dry. In these cases, in order to enable uniform germination, 40 mm of water was provided after sowing. When it was dry in June, 40 mm was again provided in the middle of the month and if needed; again, the Meteorological data are available from the Hungarian Meteorological Service (HMS) (https://www.met.hu/en, accessed 15 January 2022). For the temperature, we used data from the HMS, and for precipitation, the Kiszombor Research Station data were better because the station is about 500 m from the field and so the data were more reliable. The European corn borer was controlled by Decis 2.5 EC (deltamethrin) 1–2 times in each season.

From the naturally infected grain, several hundred fungal lines were isolated. The nucleotide sequences for species determination were as follows: *F. graminearum* and *F. verticillioides*: EF1-α primers: ef1: ATGGGTAAGGARGACAAGAC and ef2: GGARGTACCAGTSATCATGTT; *A. flavus*: CaM primers: CMD5: CCGAGTACAAGGARGCCTTC and CMD6: CCGATRGAGGTCATRACGTGG [48]. *F. graminearum* and *F. verticillioides* were dominant. As *F. culmorum* was rarely isolated from maize grains, *F. graminearum* played an exclusive role. In addition to *F. verticillioides*, a low rate of *F. proliferatum* was found. *Aspergillus* spp. *A. flavus* dominated, but *A. niger* (several strains can produce fumonisins) and *A. parasiticus* occurred rarely. Of the 87 *A flavus* lines, about half were able to produce aflatoxin on a rice medium, but only 12 could produce it under field conditions. The majority of the *A. flavus* isolates are not real aflatoxin producers. As different *Fusarium species* can produce the same toxin and each *Fusarium* spp. can produce many toxins, it is difficult to identify exactly which *Fusarium* spp. is behind a specific toxin contamination.

#### 2.1.2. Experimental Design

The test plots were sown using a complete randomized block design with three replicates [49,50,51]. From 2014 to 2020, 18–23 hybrids were sown yearly in nearly 4 m long rows, with a 75 cm row distance and 20 cm spacing between the plants. For this paper, only the non-inoculated control rows were used. As we worked with toothpick inoculation, active infection material was not expected to cause a natural infection. The ear rot mean of the natural *Aspergillus* spp. was 0.002% and that of the artificial inoculation was 0.250% (a 125-fold difference) in 2019–2020 [48]. A similar natural infection was recognized in the corn nursery, far from this experimental site. As no storage was involved at harvest, the toxin contamination could have come only from the field and so we had to consider it as of preharvest origin—useful for risk analysis when they were higher than the EU limit of 20 mg/kg [2,3,4]. This was also valid for DON and fumonisins. The meteorological conditions were highly variable (Table 2).

The experimental plots were sown at the end of April or the very beginning of May, depending on the weather.

**Table 2:** Monthly averages of temperature and the precipitation and number of hot days (above a 35 °C peak temperature) at the Kiszombor experimental station, 2014–2021.

	Mean Temperature (°C)					
	June	July	August	September	October	Mean
2014	21.1	23.1	21.9	18.4	12.9	19.5
2015	22.2	24.9	24.4	19.3	11.5	20.5
2016	21.9	22.9	21.8	19.3	10.8	19.3
2017	23.1	23.3	24.2	18.0	12.5	20.2
2018	21.6	23.6	24.6	18.9	14.3	20.6
2019	23.8	22.5	24.5	18.6	14.0	20.7
2020	21.6	22.3	23.7	19.3	12.8	19.9
2021	22.5	25.3	22.3	17.8	10.8	19.7
	Number of hot days above a 35 °C daily maximum					
	June	July	August	September	October	Sum
2014	0	0	0	0	0	0
2015	0	11	7	0	0	18
2016	1	1	0	0	0	2
2017	0	2	11	0	0	13
2018	0	0	0	0	0	0
2019	0	1	1	0	0	2
2020	0	0	0	0	0	0
2021	2	3	0	0	0	5
	Precipitation (mm)					
	June	July	August	September	October	Sum
2014	64.5	180.0	45.5	77.5	75.7	443.2
2015	7.0	19.0	123.5	35.0	94.0	278.5
2016	90.0	141.7	36.3	51.4	77.0	396.4
2017	49.4	45.4	18.8	36.0	35.4	185.0
2018	116.3	65.6	59.1	37.8	10.4	289.2
2019	111.3	47.8	23.3	30.5	27.1	240.0
2020	113.6	117.1	59.9	24.9	92.3	407.8
2021	35.1	72.8	41.1	26.9	35.9	211.8

#### 2.1.3. Harvest, Evaluation of Infection, and Sample Preparation

When the latest hybrids ripened and the grains contained 17–18% moisture, they were harvested in mid-September or later. The ears of each row were snapped and placed on the soil on a clean textile jute bag spread on the soil to avoid possible *Aspergillus* contamination from the soil. On the same or next day, each cob was inspected for visual *Fusarium* and *Aspergillus* infection and insect damage separately, measured as a percentage [52,53]. We used a much more sensitive scale for evaluation than usual, as for the toxin contamination highly precise evaluation was needed for the seasons of low infection or higher resistance. As a hybrid ear contained about 750–800 grains, one infected grain in a cob indicated a 0.15% infection. If there were 7–8 infected kernels in an ear, this would be rated as 1% coverage and so on. Above 10%, the scale was divided into 10% distances. Each year, Aspergillus isolates were collected from the grains, and their identification was made by calmodulin gene of the fungal DNA, which was amplified using the cmd5 and cmd6 primers [54]; they belonged mostly to *A. flavus*.

Just as in the case of ear rot evaluation, five corn ears with average infection severity and without insect damage were selected for toxin evaluation. The cobs were collected and dried in a dry room as soon as possible until their moisture levels were 13–14%.

The sampling process was critical [52,53] as the natural infection severity was low, seldom higher than 2%.To decrease sampling problems, the yields of the five selected ears without visible insect damage were roughly milled to 1–2 mm pieces, which were then mixed thoroughly; from the mixture, a 100 g subsample was separated. This process was performed for each replicate. The three replicates were pooled and mixed, and 100 g was separated for toxin analysis. This 100 g sample was sent to the BBT laboratory in Nagyigmand. In 2021, the procedure was the same, but from the sample, two subsamples were separated, each from 5 different places of the bag and they were subject to analysis.

### 2.2. Toxin Analysis

The analytical procedure BBVM-111:2015 was performed in the BBT laboratory. The UPLC-MS/MS method was used for common mycotoxins, accredited by The Hungarian National Accreditation Authority under the designation NAH-1-1-1560-2016 [55].All reagents and solvents were of chromatographic or analytical grade. Ammonium acetate was purchased from Thomasker Ltd., (Budapest, Hungary, www.thomasker.hu, last accessed in 18 December 2021). Solvents and glacial acetic acid (AcOH) were purchased from Honeywell™ (Speciality Chemicals GmbH, Hannover, Germany). Ultrapure water (resistivity > 18 MΩˑcm) was prepared freshly on each analysis day using a Human Corp. (Seoul, South Korea, www.humancorp.co.kr, Zeneer Power I. water purification system). Mycotoxin reference standard solutions were purchased from Romer Labs^®^Erber, Austria (www.romerlabs.com. 3430 Tulln, Austria).

As we followed the same procedure published in [48], a detailed description is not needed. The linearities were as follows: y: AFB1 y = 30321x + 101.77 R^2^= 1; AFB2 y = 28,588 + 210.42, R^2^= 1: AFG1 y = 10847x − 880.95, R^2^ =1; AFG2 y = 17,068 − 177.7 R^2^ = 1; DON y788.54 + 4060.2, R^2^ = 0.9999; FUMB1 y = 638.74 + 26.793, R^2^ = 1; FUMB2 y = 730.93 + 517.86, R^2^ = 01. The LOD data were 0.125 μg/kg for aflatoxin G2, for FUM B1 + B2 and DON 2.500, and for AFB1, B2 and G1 0.031 μg/kg, respectively. The LOQ concentrations were 0.004, 0.080, and 0.001 μg/kg in the same order. Recovery data were between 89.2% (DON) and 112.8% (FUM B_1_); the others were between them.

#### 2.2.1. Sample Preparation

The maize samples were ground using a Perten laboratory mill (Type: 3310, Perten Instruments, Hagersten, Sweden). In all, 5 g of each sample were weighed in a 50 mL PP centrifuge tube by collecting a 0.5–1 g portion from the bulk sample. Then, 40 mL of an acetonitrile–water–AcOH mixed solution (70:29:1, *v/v/v*) for AB_1_ and FB_1-2_, or ultrapure water for DON, was added as an extraction solvent. Both the sample and spiked sample vials were filled up to 700 µL using ultrapure water as a diluent for DON and an acetonitrile–water–AcOH mixed solution (20:79:1, *v/v/v*) for the other mycotoxins.

#### 2.2.2. Chromatography

A Kinetex^®^ C18 100Å UPLC (Phenomenex Inc., Torrance CA, USA, www.phenomenex.com, Last accessed on 18 December 2021) column (2.1 × 100 mm, 1.7 μm) was kept at the constant temperature of 30°C. The flow rate was 0.35 mL min^1^, and a 5 µL partial loop injection was used (loop of 20 µL). Mobile phases were buffered with 5 mM ammonium–acetate. Ultrapure water with 1% AcOH and 4% MeOH was used as mobile phase A, and MeOH containing 1% AcOH with 2% ultrapure water was used as mobile phase B. The gradient was programed for the negative mode as follows: 0–5 min A: 100%, 5–8 min A: 10%, 8–10 min A: 10%, 10–10.5 min A: 100%, and 10.5–12 min A: 100%. For the positive mode, the gradient was programed as follows: 0–2 min A: 95%, 2–6 min A: 5%, 6–12 min A: 5%, 12–12.5 min A: 95%, and 12.5–14 min A: 95%.In the samples collected in 2014–2020, among the aflatoxins, only AFB1 was measured, but in the samples collected in 2021, all four were analyzed, but only sporadic and low AFB1 was found.

#### 2.2.3. MS/MS Analysis

The main mass spectrometer parameters were as follows: source temperature 350°C, curtain gas 40 psig, ion source gas 35 psig, detector voltage 5 kV, and entrance potential 10 V. The collision gas was set to medium. MS/MS conditions were optimized in the Analyst^®^1.6.2 Compound Optimization module (Sciex Inc., Framingham, Connecticut U.S. www.sciex.com, Last accessed on 18 December 2021) using direct infusions of each analyte standard. DON was measured in the negative mode, while the other mycotoxins were measured in the positive mode. Raw results were calculated using the Analyst^®^1.6.2 software package. The concentration was calculated as c_spike_ = c_stock_ˑ(V_spike_ ÷ V_final_). The final results were corrected with the recovery of the standard addition; the sample was weighed, and the extraction solvent added using the following equation: c_sample_ = (c_raw_ ÷ V_extr_ ÷ m_sample_) * ((c_rawspiked_ – c_raw_) ÷ c_spike_).

### 2.3. Statistical Methods

For the visual ear rot data, a two-way ANOVA model was used without replicates. For the toxin data in the 2017–18 and 2019–20 tests, a two-way ANOVA model was applied without replicates. Variance was assessed via a one-way ANOVA. In this way, the year effect could be neutralized to some extent and also the within value decreased. In addition, regression and correlation tests were used. We also compared ear rot and toxin data. For the risk analysis, we used the EU toxin limits. The mycotoxin data from the two subsamples were analyzed by a two-way ANOVA in 2021. The statistical methods were based on Sváb [56] and Weber [57] as well as on the built-in Excel functions.

## 3. Results

The yearly data are presented in Tables S1–S8). The ANOVAs of the *Fusarium* and *Aspergillus* visual ear rot data are not shown; only the LSD 5% values are provided in the STables. The *Fusarium* ear rots showed significant genotype differences each year. However, the sporadic and low *Aspergillus* ear rot data were not significant in any of the experiments performed. The ranking in the tables was made according to the DON contamination.

### 3.1. Natural Ear Rot and Toxin Contamination of Hybrids, 2014–2021

For each STable, we present the toxin production data for a percentage of visual infection. This was necessary to demonstrate this difference between hybrids, which significantly influences the food safety risk of the given hybrids. The many-fold deviations show the hybrid differences. The empty cell shows the presence of the zero divisors.

In 2014, the *Fusarium* ear rot varied between 0.3% and 2.9%, but in extreme cases, the hybrid differences were highly significant, and in spite of the low rated ear rot, a high toxin contamination was observed (Table S1). The year 2014 was not an aflatoxin-prevalent year, but in six genotypes, at least 20 μg/kg or higher contamination levels were observed, which is over the EU limit of 20 μg/kg. Only one hybrid was found in which toxin contamination was lower than the EU limit (suggested feed limits: DON swine 0.9, FUM 5 mg/kg, and aflatoxin 20 μg/kg). The conclusion is that the ecological conditions allowed for the highly variable toxin contamination.

The year 2015 was quite different (Table S2). The mean concentration of *Fusarium* in the ear rot data was 1.21% and was close to the mean infection severity in 2014. However, that of Aspergillus was only 0.04%, but the toxin contamination severity was much lower. Eight hybrids were found with lower data than average for each toxin. In the others, significantly very different combinations of the low and high values were found.

The year 2016 provided different values (Table S3). The *Fusarium* infection severity was significantly lower than that in 2014 and 2015. AER was found only in one hybrid. A DON epidemic was not observed, only one hybrid had a DON value above the limit. Fumonisins were identified in each hybrid, but only two showed fumonisin levels above the EU limit. There was no aflatoxin epidemic as all hybrids showed aflatoxin levels lower than 20 μg/kg. However, even though the hybrids were found to have generally low toxin levels, they were not fit for use in piglets as they did not adhere to the needs criteria for baby food quality.

In 2017 (Table S4), the mean *Fusarium* visual infection rate was low again, with all values being lower than 1.0%, ranging between 0.28% and 0.82%. The *Aspergillus* ear infection was also low (mean 0.04%). For DON, only 2 genotypes had DON values higher than 0.9 and, in 15 cases, no DON was found to be above the detection limit (0.08 mg/kg). For all fumonisins, except 1, all genotypes had fumonisins below the limit, but 13 produced higher fumonisins than the calculated piglet limit. For aflatoxin, the results were different. Only 11 had aflatoxin levels below the limit, but 12 had aflatoxin levels far above the limit, with the highest value of 385 μg/kg. Only four hybrids were applicable for piglets. Only 6 hybrids had visual symptoms in addition to AFB1, and 17 hybrids did not show visual symptoms, but all presented more or less aflatoxin contamination. This finding is important as seemingly healthy hybrids can contain high concentrations of aflatoxin, similar to the highest contaminated KK 4420 hybrid.

The data for 2018 were variable, with low visual *Fusarium* infection and no genotypes showing *Aspergillus* infection (Table S5). For DON, six hybrids surpassed the adult pig limit, one surpassed the piglets 0.2 mg/kg limit, and all others were of baby food quality. No fumonisin epidemic was observed, all genotypes had fumonisin lower than 5 mg/kg, and a further six could not be used for the piglet feed. It can be said that the aflatoxin situation was good, but we had two heavily contaminated hybrids with no visual symptoms (70 and 190 μg/kg). The situation is similar for Koregraf and Korimbos, where, at nearly zero, natural Fusarium ear rot yielded a DON contamination level of 4.20 and 9.90 mg/kg. Therefore, also for DON, there can be cases where there are no symptoms or the symptom severity is low, but the toxin contamination is high.

Both Fusarium and Aspergillus ear rots were very low in 2019 (Table S6). However, 11 hybrids had a much higher DON content than the limit of 0.9 mg/kg, and a further 5 were not suitable for the piglet feed. For fumonisins, all hybrids had lower values than the 0.9 mg/kg limit, but 11 hybrids contained a higher fumonisin content than that which we would suggest for piglets. Two hybrids had a significantly higher aflatoxin level than the adult swine limit, again without visible Aspergillus infection.

Another epidemic situation was observed in 2020 (Table S7). The Fusarium visual infection remained sporadic and low. This was also the case for Aspergillus spp., where 12 hybrids remained symptomless. For DON, all hybrids had lower levels than 0.9 mg/kg, except two; all were suitable for the piglet feed. Fumonisin concentration, except Koregraf, remained lower than 5 mg/kg. For piglets, however, 10 hybrids had higher contamination levels than those suggested (1 mg/kg, 20% of the adult limit of 5.0 mg/kg EU limit). In this respect, the data are not very good. As for aflatoxin, 13 hybrids had values lower than 20 μg/kg and 5 hybrids had very high levels (227–2286 μg/kg), the highest numbers that we observed in these 8 years. In four of the cases, no visual infection was connected to these values.

The year 2021 was different (Table S8). Fusarium ear rot was minimal (mean 0.09%) and it was the first year in which Aspergillus infection was zero for all hybrids. For DON, except for two hybrids, all had toxin contamination levels below the swine EU limits. In addition, 16 hybrids had lower toxin contamination levels for all mycotoxins tested. Thus, two subsamples were analyzed for all pooled replicates. We received highly significant differences in all hybrids. For DON, the variation is 4.77 mg/kg and for LSD 5% is 0.13, and the rate between them is 36.6-fold. For fumonisin, it is 34.5-fold and, for aflatoxin, 14.28-fold. This means that, even at not too high toxin contamination levels, the relatively small differences are mostly significant.

In the 8 years during which this study was conducted, we saw relatively low or very low natural infection severity. At these low infection severity levels, extremely high toxin contaminations were recorded, far above the EU limits. It was often the case that, at these low infection severity values, extremely high toxin contaminations were recorded, far above the EU limits. Therefore, we should consider these values as serious risk factors.

### 3.2. Correlations between Natural Ear Rot Infection Severity and Natural Toxin Contamination

For each year, the correlations between visual ear rot and toxin contamination were calculated (Table 3). In 4 years, no significant correlations were found; in 3 years, one of eight correlations was significant, and in 2017, two of eight correlations were significant. A correlation that was valid for all relations was not found, indicating that many contradictions were found in the individual years. As these were seen each year, it is not an accident, but a characteristic of the situation. Therefore, on the basis of the visual ear rot severity data, no useful forecasts can be drawn about the possible toxin contamination. Every year, hybrids were found with higher than the EU-suggested toxin contamination levels. This is a risk factor as such grain cannot be sold at all or cannot be sold at a full price. Therefore, farmers consider this a serious problem. Among the 24 possible correlations (DON/FUM, DON/AFB1, and FUM/AFB1), only two were significant, but this result was not valid for the other relations in the given year. This means that low infection severity values do not automatically indicate low toxin contamination and vice versa. There is another factor that is important. When DON contaminations are below the limit, this does not mean that high FUM or aflatoxin contamination could not be present. For this reason, all three toxins should be measured. This underlines the necessity of multitoxin analyses. Lastly, hybrids in given years react very differently to the same natural infection conditions. The extent to which this indicates resistance differences will be an important question addressed in the Discussion Section.

The hybrids also changed to some extent from year to year, and their yearly means showed considerable differences (Table 4). Over the 8 years, the mean severity of ear rot was low. Even in the worst epidemic year (2014), the mean visual ear rot level on unshelled ears was just above 1%. On the basis of Table 5, we could identify two epidemics for DON (2014 and 2019), two epidemics for FUM (with strong variation within years), and three epidemics for aflatoxin (2015, 2017, and 2020). In 2016, 2018, and 2021, no toxin caused an epidemic, but here too, one or more hybrids were found every year with higher toxin contamination. Aflatoxin occurred every year, so we can consider its regular occurrence normal in the South-east part of the country.

Of the tested hybrids, Korimbos and Valkür were investigated for the longest time. The resistant control Korimbos (Table 5) suffered three epidemics for DON (2014, 2018, and 2019). The resistance to toxin contamination differed. Aflatoxin showed epidemics in 2017 and 2020. Fumonisins were more severe in 2014 and 2016. Valkür (Table 6) exhibited two DON epidemics (2018 and 2019) in the 5 years but no epidemic of fumonisin and aflatoxin. This is considerably better than the values from Korimbos; Valkür is more stable and less susceptible to yearly environmental fluctuations in deterring aflatoxin production and/or accumulation.

**Table 5:** Natural and visual ear rot infection severity and toxin contamination of the resistant control Korimbos, 2014–2020.

Year	Fus. Visual Ear Rot	Asp.Visual Ear Rot	Toxins		
	%	%	DON (mg/kg)	FB_1_ + B_2_ (mg/kg)	AFB_1_ (mg/kg)
2014	0.72	0.000	**2.61**	**7.09**	0.000
2015	1.47	0.330	0.00	0.81	0.000
2016	0.11	0.090	0.00	**2.03**	0.001
2017	0.18	0.000	0.60	0.83	**0.055**
2018	0.06	0.000	**4.20**	0.25	0.000
2019	0.05	0.000	**6.80**	1.39	0.000
2020	0.10	0.000	0.17	0.22	**0.816**
2021	0.04	0.000	0.14	0.28	0.010
Mean	0.34	0.050	1.82	1.61	0.110

Bold: Toxin data above the EU limit, it signalizes epidemic occurrence of toxins.

### 3.3. Natural Toxin Contamination in Two Years’ Trials, 2017/2018 and 2019/2020

In Table 7, the 2017/2018 data show the yearly differences in toxins and their mean values in 18 hybrids, which are ranked by the mean values of DON. For DON, the difference is 6-fold between the 2 years, for fumonisins the difference is nearly 3-fold, and, for AFB1, the difference is 13.6-fold. Significant genotype differences were only found for FUM, and the yearly mean values were the closest here. This is the result of changing weather conditions between years. No significant correlations were found between years, except for fumonisins (r = 0.76; *p* =0.001). There was a highly significant correlation between the fumonisin mean and the 2 years separately. However, we found five hybrids (DKC 5830, P 9441, PR37F80, DKC 4717, and P 9903) with a low risk in all years and toxins that showed the possibility of selecting low-risk hybrids from the registered hybrids (shown in bold in Table 9). The lesson is that high toxin values always signal a risk. We found highly significant correlations between the mean and the yearly data with a larger toxin contamination column and closer mean values were found for both years, which correlated more positively with the mean values.

Table 8 summarizes the two-year data from 2019 and 2020. In FUM, we found the closest mean values, but the genotype differences were not significant for any toxigenic species. For this, opposite reactions were responsible in the 2 years, as in the case of Koregraf and the ES Lagoon. No significant correlations between two yearly averages were significant; this is similar to what is shown in Table 7. No epidemic was recorded for DON in 2020; only traces of DON contamination were found. This was also the case for aflatoxin B_1_. The correlations between means and the higher contaminated year were above r = 0.90. From the lower contaminated years, no significant correlations were found; this differed from the results of Table 5. Three hybrids (ES Harmonium, SY Zephyr, and Kathedralis) were selected with lower toxin contamination than average for the toxins and years. P 9718E was close to them; here, only DON 2020 0.13 was slightly worse than the mean, but even so without harmful DON contamination.

For both experiments, large differences were found across hybrids in terms of the contamination levels of each toxin each year. Further, for many hybrids, the responses in the 2 years were different, but some hybrids were also identified with low toxin contamination in both years. In 2020, this was not a problem for DON, but it was for fumonisins. All possible cases occurred. For aflatoxin B_1_, except for Lagoon, every hybrid had contamination levels close to zero, but 2020 showed vastly different numbers.

**Table 7:** Natural preharvest toxin contamination levels in maize hybrids, 2017–2018; ranking: DON.

Hybrid	2017	2018	Mean	2017	2018	Mean	2017	2018	Mean
	DON (mg/kg)			FUM B_1_ + B_2_ (mg/kg)			AFB1 (μg/kg)		
4517	0.00	0.0	**0.00**	10.21	3.8	**6.99**	58	0.0	**29.00**
DKC 5542	0.00	0.0	**0.00**	4.34	1.1	**2.74**	66	0.0	**33.00**
**DKC 5830**	0.00	0.0	**0.00**	0.18	0.0	**0.09**	5	0.0	**2.50**
**P 9241**	0.00	0.0	**0.00**	1.42	0.0	**0.71**	9	19.0	**14.00**
P 9537	0.00	0.0	**0.00**	1.85	1.2	**1.55**	22	0.0	**11.00**
P 9911	0.00	0.0	**0.00**	2.09	0.3	**1.20**	21	0.0	**10.50**
**PR37F80**	0.00	0.0	**0.00**	1.22	0.6	**0.89**	33	5.0	**19.00**
DKC 4541	0.00	0.1	**0.04**	2.53	0.4	**1.46**	17	0.0	**8.50**
Szegedi 521	0.09	0.0	**0.05**	0.52	0.8	**0.66**	169	12.0	**90.50**
**DKC 4717**	0.00	0.1	**0.07**	1.24	2.6	**1.94**	14	1.0	**7.50**
**P 9903**	0.00	0.2	**0.09**	0.83	0.0	**0.42**	5	0.0	**2.50**
DKC 4590	0.37	0.0	**0.19**	2.22	1.3	**1.77**	2	1.3	**1.66**
Cardixxio Duo	0.39	0.0	**0.20**	0.36	0.0	**0.18**	4	1.0	**2.50**
DKC 4943	0.14	0.5	**0.32**	0.76	0.1	**0.45**	43	0.0	**21.50**
Valkür	0.22	1.0	**0.61**	0.81	0.2	**0.51**	10	0.0	**5.00**
Fornad	0.00	2.1	**1.04**	2.88	0.3	**1.59**	7	6.0	**6.50**
Siló Star	0.00	2.6	**1.31**	0.53	0.1	**0.32**	77	0.0	**38.50**
Korimbos	0.60	4.2	**2.40**	0.83	0.3	**0.54**	55	0.0	**27.50**
Mean	0.10	0.60	**0.35**	1.93	0.73	**1.33**	34.28	2.52	**18.40**
LSD 5%			ns			2.31			ns
ns = non-significant; **bold numbers**, means for two years, 2017–2018; **bold names**: hybrids lower than the mean in all years and toxins.									
Correlations	2017	2018	Mean	2017	2018	Mean	2017	2018	
	DON (mg/kg)			FUM B_1_+B_2_ (mg/kg)			AFB1 (μg/kg)		
DON 2018	0.4819 *^a^								
DON Mean	0.5861 *^b^	0.9923 ***							
FUM 2017	–0.2426	–0.1784	–0.1990						
FUM 2018	–0.1784	–0.2591	–0.2646	0.7628 ***					
FUM Mean	–0.2352	–0.2139	–0.2309 ^b^	0.9782 ***	0.8803 ***				
AFB1 2017	–0.0265	0.1374	0.1233	0.0797	0.1517	0.1071			
AFB1 2018	–0.1654	–0.1401	–0.1528	–0.1300	–0.1647	–0.1482	**0.2414**		
AFB1 Mean	–0.0456	0.1154	0.1003	0.0611	0.1262	0.0853	0.9930 ***	**0.3540**	
*** *p* = 0.01; * *p* = 0.05; ^a^ **bold**: correlations between 2017 and 2018; ^b^ numbers in bold and italics show correlations between yearly data and means.									

**Table 8:** Natural preharvest toxin contamination levels in maize hybrids, 2019–2020; ranking: DON.

Hybrid	2019	2020	Mean	2019	2020	Mean	2019	2020	Mean
		DON (mg/kg)			FUM B1 + B2 (mg/kg)			AFB1 (μg/kg)	
Koregraf	0	0	**0**	0.36	10.9	**5.63**	0	703	**351**
ES Lagoon	0	0	**0**	2.28	0.88	**1.58**	21	18	**19**
P0725	0	0	**0**	0.63	4	**2.32**	0	1588	**794**
Sy Zoan	0	0	**0**	2.54	2.17	**2.36**	0	0	**0**
Illango	0.09	0	**0.05**	1.89	1.57	**1.73**	0	2286	**1143**
**ES Harmonium**	0.25	0	**0.13**	0	0.59	**0.3**	1	6	**3**
**SY Zephir**	0.33	0	**0.17**	0	0.59	**0.3**	0	6	**3**
**Kathedralis**	0.53	0	**0.27**	0.43	1.9	**1.17**	0	0	**0**
Kleopatras	0	0.7	**0.35**	3.34	1.27	**2.31**	0	227	**113**
P9415	1.16	0	**0.58**	1.35	0.26	**0.81**	0	0	**0**
P9718E	1.13	0.13	**0.63**	0.24	0	**0.12**	0	8	**4**
Sy Talisman	1.04	0.4	**0.72**	1.07	2.68	**1.88**	2	2	**2**
Valkür	2.4	0.15	**1.28**	0	3.3	**1.65**	0	0	**0**
Konfites	3.27	0.12	**1.7**	1.23	3.22	**2.23**	0	4	**2**
DKC 5830	4.35	0	**2.18**	2.84	3.28	**3.06**	0	0	**0**
Armagnac	4.65	0	**2.33**	0.61	0.88	**0.75**	0	18	**9**
Korimbos	6.8	0.17	**3.49**	1.39	0.22	**0.81**	0	816	**408**
DKC 4541	7.18	0	**3.59**	0	1.08	**0.54**	0	0	**0**
Mean	1.84	0.09	**0.97**	1.12	2.16	**1.64**	1.33	316	**158**
LSD 5%			**ns**			**ns**			ns
Correlations		DON (mg/kg)			FUM B1 + B2 (mg/kg)		AFB1 (mg/kg)		
	2019	2020	Mean	2019	2020	Mean	2019	2020	
DON 2020	–0.081 ^a^								
Mean	0.997 ***^b^	–0.004							
FUM 2019	–0.120	0.391	–0.090						
2020	–0.205	–0.092	–0.212	–0.093					
Mean	–0.244	0.071	–0.239 ^b^	0.318	0.914 ***				
AFB1 2019	–0.208	–0.092	–0.216	0.258	–0.130	–0.019			
2020	–0.174	–0.112	–0.183	0.098	0.213	0.243	–0.132		
Mean	–0.176	–0.112	–0.185	0.1	0.212	0.243	–0.124	0.999 ***	
*** *p* = 0.001; ^a^ number in bold show correlations between 2019 and 2020; ^b^ numbers in bold and italics show correlations between yearly data and means.									

### 3.4. Toxin Production for 1% Ear Rot Coverage

During the 8 years we chose for the study, we mentioned cases where the same or similar ear rot presented highly different toxin data for each toxin. The rates of toxin production for a percentage of ear rot were calculated, and some regularity in the data could be detected (Table 9). The genotype differences for toxin contamination for a percentage of visual infections were exceptionally larger. The yearly data are presented in Tables S1–S8. These 18 hybrids were tested in 2017–2018 as well as 2019–2020. The first four hybrids are shown and were tested in each year from 2017 to 2020. Korimbos seems to have a high toxin production capacity for all three toxins. Larger values were obtained for aflatoxins. The large differences between hybrids are clear, but not the reasons. As this is a new aspect of the toxin syndrome in maize, at present, no genetic explanation is possible. For this, however, artificial inoculation methods should be used, as the experimental conditions can be better controlled.

**Table 9:** Toxin production of the hybrids for a unit (%) of visual ear infection rate, 2017–2020.

Hybrid	2017–2018			Hybrid		2019–2020	
	DON	FUM B_1_ + B_2_	AFB1		DON	FUM B_1_ + B_2_	AFB1
	(mg/kg)	(mg/kg)	(μg/kg)		(mg/kg/%)	(mg/kg/%)	(μg/kg/%)
DKC 4541	0.02	0.71	4.15	DKC 4541	12.0	1.8	0.0
DKC 5830	0.00	0.14	4.01	DKC 5830	13.2	18.5	0.0
Korimbos	6.75	1.52	77.46	Korimbos	46.5	10.7	5440.0
Valkür	7.63	6.31	62.50	Valkür	24.3	31.4	0.0
DKC 4590	0.12	1.17	1.10	ES Harmonium	0.5	1.2	14.1
DKC 4717	0.06	1.87	7.25	ES Lagoon	0.0	8.4	104.0
DKC 4943	0.33	0.47	22.40	Illango	0.3	9.7	6439.4
DKC 5542	0.00	2.47	29.73	Kathedralis	1.2	5.5	0.0
Cardixxio Duo	0.25	0.23	3.21	Kleopatras	2.2	14.2	698.5
Fornad	0.55	0.84	3.45	Konfites	8.3	10.9	9.8
P 9241	0.00	1.04	20.44	Koregraf	0.0	58.2	3636.2
P 9537	0.00	2.05	14.62	P0725	0.0	13.4	4602.9
P 9903	0.06	0.32	1.91	P9415	2.5	3.5	0.0
P 9911	0.00	0.81	7.12	P9718E	7.0	1.3	44.4
PR37F80	0.00	0.51	10.89	Sy Talisman	2.7	7.0	7.5
Siló Star	2.14	0.52	63.11	SY Zephir	1.0	1.9	18.9
Szegedi 521	0.04	0.52	71.83	Sy Zoan	0.0	13.3	0.0
4517	0.00	3.88	16.11	Armagnac	17.2	5.5	66.7
Mean	1.00	1.41	23.40		7.71	12.03	1171.25

Four hybrids were tested between 2017 and 2020 (Table 10). In 2018 and 2019, Korimbos showed higher contaminations of DON than the limit. This is why we should be careful when using the data to determine resistance differences. The ANOVA model was a two-way model without replications, so the yearly differences could be neutralized to some extent. In 2019, both DKC 5830 and DKC4541 had high DON levels. In other years, the data were very low or zero (lower than the detection limit). Due to the high yearly variation, the hybrid mean values did not show resistance differences. We also found a similar situation in fumonisins and aflatoxin B1. We may not be able to generalize these statements for every hybrid. For DON, Valkür provided the most stable performance, with a variance of 1.09; DKC 4541 showed the highest variance (instability) (12.77). Korimbos proved to be the most stable (variance 0.29) for FUM, and the maximum was 3.28 for DKC 5830. For AFB1, DKC 5830 had the lowest mean performance and variance (6), showing a high stability, but the worst was Korimbos, with a variance of 159740. This also shows that the data do not support the idea of a general toxin resistance as being valid for all toxins and hybrids.

Natural infection is influenced by weather conditions, a fact which poses interesting questions regarding the expected effects of climate change. To examine this, we compared toxin contamination in the naturally infected controls with the most relevant weather data. The correlations between disease severity, toxin contaminations, and meteorological data show a complicated picture (Table 11). The natural Fusarium infection correlated significantly with DON and FUM B_1_ +B_2_ concentration, indicating the presence of both *F. graminearum* and *F. verticillioides*. The DON/FUM correlation is surprisingly close, but the significance of *F. verticillioides* seems to be larger, as the natural infection itself did not have a significant relationship with DON.

The monthly temperature means did not show a significant relation with infection severity and toxin contamination. Therefore, temperature alone is not sufficient to cause disease or toxin epidemics. The considerable number of hot days above 35 ^o^C inhibited Aspergillus infection in June, in July the Fusarium infection remained neutral, but for Aspergillus, we found a similar response as that in June, and this was just below the limit. However, the increase in hot days increased fumonisin concentration significantly. The higher number of hot days in August increased natural Fusarium infection. These days in September strongly increased DON and fumonisin contaminations, and all other correlations were not significant. The high precipitation decreased Aspergillus symptoms in June and, in July, it nearly reached significance in DON; the rain increased fumonisin contamination in July. The high September and October precipitation strongly increased DON and fumonisin contaminations and no other correlations were significant. In several cases such as in October, AFB1 concentration was close to significant. Comparing the three weather traits, the September hot days and precipitation strongly increased DON and FUM concentrations.

**Table 11:** Correlation coefficients between meteorological monthly means and the yearly averages of hybrids for natural symptom severity and toxin contamination.

Temperature: Mean Data for Traits and Months					
Traits	Fus^x^%	Asp%	DON (mg/kg)	FUM B1 + B2 (mg/kg)	AFB1 (μg/kg)
Asp%	0.481				
(mg/kg)	0.481	−0.335			
(mg/kg)	0.673 *	−0.116	0.927 *		
(μg/kg)	−0.099	0.110	−0.290	−0.105	
June	−0.393	0.279	−0.350	−0.527	−0.256
July	0.262	0.312	−0.179	−0.112	−0.335
August	0.033	0.459	−0.410	−0.475	0.200
September	0.172	−0.016	−0.280	−0.123	0.452
October	0.007	−0.193	0.291	0.081	0.038
Mean	0.080	0.351	−0.270	−0.403	−0.025
No. of hot days above 35 °C: Mean data for traits and months					
Traits	Fus%	Asp%	(mg/kg)	(mg/kg)	(μg/kg)
June	−0.467	−0.343	−0.209	−0.254	−0.310
July	0.535	0.699	−0.315	−0.122	−0.006
August	0.340	0.962	−0.305	−0.147	0.008
September	#	#	#	#	#
October	#	#	#	#	#
Sum	0.450	0.938	−0.383	−0.185	−0.032
Precipitation mm, Mean data for traits and months					
Traits	Fus%	Asp%	(mg/kg)	(mg/kg)	(μg/kg)
June	−0.535	−0.656 *	0.022	−0.175	0.192
July	0.038	−0.595	0.603	0.650 *	0.084
August	0.625 *	0.391	−0.170	0.051	0.293
September	0.578	−0.177	0.817 ***	0.874 ****	−0.370
October	0.424	0.199	0.052	0.363	0.570
Sum	0.251	−0.445	0.454	0.586	0.351
**** *p* = 0.01; *** *p* = 0.02; * *p* = 0.10; ^x^ Fus = Fusarium; Asp = Aspergillus.					

## 4. Discussion

### 4.1. Mycotoxins and Their Preharvest Character

Aflatoxin B_1_ occurred every year. The maximum value varied greatly every year but was only lower than the EU limit of 20 μg/kg in 2016. In 2014, it was 121 μg/kg; in 2015, it was 1030 μg/kg; in 2017, it was 385 μg/kg; in 2018, it was 70 μg/kg; in 2019, it was 65 μg/kg; and in 2020, it peaked at 2286 μg/kg. The yearly means were higher than 20 μg/kg in 2015 (87 μg/kg), 2017 (51 μg/kg), and 2020 (316 μg/kg). These results are significantly higher than the EU limits [2,3,4]. Therefore, in Hungary, aflatoxin B_1_ should be considered a regularly occurring mycotoxin in maize before harvest. This is also true for the mean values of DON (epidemics in 2014 and 2019) and fumonisins (epidemics in 2014 (20.79 mg/kg) and 2015 (4.03 mg/kg) but not in 2016 (2.16 mg/kg). Different weather patterns induce different epidemics; in some years, more rain can increase toxin contamination. Genotype differences seem to be large. We do not discuss resistance differences, but it is possible that these differences could be an explanation. This agrees well with the conclusions of Munkvold and White [7]. This does not mean that postharvest control is not of great significance [24]. On the contrary, it is. However, excellent quality at harvest must be preserved during a long storage period. The conclusion is that, without effective preharvest control methods, the problem cannot be solved. The weather data and the mycotoxin contamination show a loose, mostly non-significant, correlation matrix. In most years, the same weather conditions allowed very large toxin differences and the differences were from toxin to toxin. Aflatoxin accumulation prefers high temperatures. In 2020, when the temperature did not reach values above 35 °C on any day, the largest aflatoxin contamination was recorded for several hybrids, and for others, the largest aflatoxin contamination was recorded for several hybrids. For others, the AFB1 content was below the detection limit. We do not think that meteorological data are not important; many other traits influence toxin contamination and, therefore, the resistance differences should also be considered responsible for the results.

One conclusion seems to be that the resistance to toxin accumulation for all three toxins and for most of the hybrids differs significantly and this can cause the highly different toxin production rates for a percent of visual infection. For a hybrid, the rates for different toxins in different seasons can also differ significantly. At present, we can only say that it is hardly possible to forecast toxin contamination on the basis of visual symptom severity alone; therefore, all samples should be tested for toxins.

### 4.2. Reasons for Controversial Visual Ear Rot and Toxin Data

Rachis can play a significant role in the extension of infection and aflatoxin contamination. *A. flavus* can infect the whole depth of the ear [58,59,60] and may invade the kernels through the rachilla [61]. Rachis resistance is, therefore, also considered a component of ear rot resistance [60]. Such a situation was found in *F. graminearum* in 2014 in Hungary. The germ part was severely infected, but the dent part was mostly healthy. The reason is that the rachilla contains 12–20% more water than the grain at different developmental stages [62]. As fungal growth on the ear surface stops at 23% grain moisture [1], on the surface of the cob (rachilla), its growth is possible for about 2 weeks longer depending on ecology, drydown, and genetic factors. Additionally, the fungus spreads at a higher speed in susceptible rachises, as compared to more resistant rachises [59]. When examining unshelled ears, such an infection remains hidden. The systemic infection of a maize plant by *A. flavus* could be one explanation for the presence of aflatoxins in symptomless ears, as aflatoxins might translocate within the plant [63]. Drought and high temperatures are almost always initiators of aflatoxin outbreaks [64], even when existing infections cannot spread [21]. Drought stress indicates proline accumulation [65], which enhances aflatoxin production [66].

**Figure 1:**
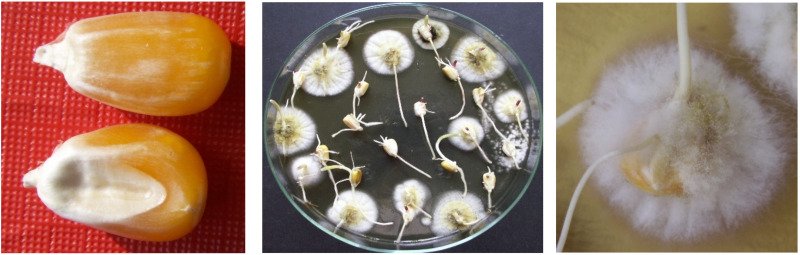
*A. flavus*-contaminated symptomless grains (left) and colonies (middle and right) developing from them following surface sterilization by a NaClO 1% solution (5 min) (courtesy of Mesterhazy, 2012).

Another source can be the seemingly healthy grain that cans experience severe *Aspergillus* infection (Figure 1, left). In such grains, a high AFB1 can be present in healthy-looking whole kernels, which could lead to a better understanding of the source of high toxin contamination without a visually detectable infection. As atoxigenic lines also occur among the *A. flavus* strains, their presence does not automatically indicate aflatoxin contamination [67,68]. These data support the view that disease and toxin regulation, even though they have common features, can be contradictory.

The correlations between symptoms and toxin contamination are mostly weak. The connection between symptoms and toxin content is better in artificial inoculation [52,53]. We have to relate the visual symptom severity found in this paper (0–2%) to the official maize hybrid tests from 2010 where the maximum *Fusarium* ear rot incidence (%) was 85% in the observed ears and 27% ear coverage. The most resistant had 27% incidence and 11% coverage (52). Furthermore, it can be seen that toxin contamination for 1% visual ear infection can be variable in different hybrids, different pathogens, and different years. We do not know much about the effects of genetics and the environment, so this is a major research objective for the future. Arid areas increase the danger of aflatoxin [69] and fumonisin [70] contamination. Rainy periods can also increase toxin contamination, as in this study, when the rainy months of September and October favored severe toxin contamination by DON and FUM but less so by AFB1.This was also observed for *Fusarium*; it seems that toxin and disease regulation do not necessarily agree [43,52,53]. Our data working with the yearly means show mostly not significant correlation among temperature, hot days, and precipitation. In the eight years of the study’s duration, only two years (2015 and 2021) had lower mean values than the EU limits for all three toxins; three epidemics were caused by DON, two by fumonisins and two by aflatoxin. In addition, a very high variability was detected between hybrids in the different years. This explains why the low natural infection below 1–2% does not provide information about suitability for food and feed safety, and sometimes high toxin contamination can be found where there is no visible infection, which is mostly characteristic for *A. flavus*. We agree that the relationships between symptoms and toxin contamination are poorly understood [34]. Our conclusion is that, with a lack of close correlations between symptoms and toxin contamination for natural infection regimes, the measurement of the toxin is the only way to receive reliable information about the food and feed safety value of a given maize lot. An indirect way to estimate toxin contamination based on natural infection does not seem reliable. As there may be many toxins in a sample, multitoxin tests are recommended.

There is another source for the mostly non-significant correlations. We showed that, for artificial inoculation tests [48], the toxin production for a percentage of visual infection can have very high differences and can lead to toxin overproduction and underproduction [48]. From this paper, it seems that this is true also for the natural infection regime.

### 4.3. Toxin Forecasting

The data clearly show that, in most cases, the correlations between visual ear rot and toxin contamination are not significant. For this reason, it is not possible to estimate toxin contamination on the basis of visual infection data. As in most cases, multitoxin contamination occurs, making the problem even more complicated. The forecasting of toxin contamination is a complex activity [38,45,46,48,69,70,71,72,73,74,75,76], and the resistance level is not included as an influencing factor. As data about resistance differences have been proved for all three pathogens [43,52,53,77,78,79,80,81], it is clear that, compared with natural data regimes, the same ecological nursery conditions result in highly different infection rates and toxin data. This clearly indicates that resistance data should be considered. There is a problem, as such data do not exist (Battilani, personal comm. 2019).Therefore, forecast scientists cannot be blamed. They simply do not have support from the various governmental organizations or plant breeders. Additionally, looking at the general occurrence of multitoxin presence in most corn samples, we need forecasting programs that can handle the three or four most important toxins at the same time. Our conclusion is that it is better to focus on the toxin contamination directly as a useful result since toxin contamination from infection data is not possible with the present knowledge. Therefore, the forecast procedure correctly concentrates on the toxins and not the symptoms. The paper showed evidence that the decision to focus on toxin forecasting was correct. For this reason, not only resistance to disease, but also a toxin regulation in hybrids being independent from resistance, should also be considered.

### 4.4. What Is the Usefullness of Natural Toxin Data?

All toxin regulations refer to natural toxin contamination [2,3,4], independently from their origin, preharvest, postharvest, or combined.

1. The entire food and feed industry is based on toxin contamination data. For this reason, the preharvest toxin data have a much higher significance than is often thought [43]. As no toxin data can be forecast from visual scores, toxins should be measured. We stress the significance of the preharvest toxin data as these provide the first possibility to act. A rapid test should be performed for every truck from the field to separate shipments with excellent quality from low-quality ones and store them later separately. Cooperativa Agraria (Guarapuara, Brazil) work according to this rule, treating more than one million tons of grain yearly (Mesterhazy, 2022, pers. communication). We have a similar experience in Hungary (Bonafarm Inc., Dalmand, Hungary) at a smaller size.

2. As hybrids arrive from many trucks, their toxin data are very useful for the grower to withdraw hybrids from production where the rate of highly contaminated lots are more frequent. This must be treated as a risk factor.

3. The mixing of grain from different hybrids and fields with different toxin contamination happens often deteriorating the entire storage content. By separate storage, this problem can be avoided. From a highly inhomogeneous grain mass, a reliable toxin contamination level is not possible, and even five-fold differences among the regular sampled muster occur (Tanyi, 2015, personal communication).

4. If growers receive feedback quickly about the value of the variety, risky cultivars can be withdrawn from production, as is the case with Cooperativa Agrária Agroindustrial, Guarapuara, Brazil. Preharvest data can provide information about the various epidemics. Based on data on natural toxin contamination, the breeders can decide whether a breeding program should be taken forward considering the pathogen and its toxin(s). At the same time, the breeders can have feedback regarding whether the hybrid they produced fits to the resistance class that was previously suggested.

5. Regular preharvest toxin controls can contribute to identifying the location, amount, and quality of maize lots in silos, which could be marketed within the country or be exported, and can provide information about the losses caused by mycotoxins.

### 4.5. Adaptation to Environmental Stresses

Climate models forecast variable warming scenarios with locally lower or higher precipitation levels [36]. There are many types of *Fusaria*-causing ear rot [82,83], but the two most damaging are *F. graminearum* and *F. verticillioides*. Resistance to them is not connected and supposedly their present significance is moderate, but the species structure may change. Therefore, the *Fusarium* spp. population structure should be checked to identify emerging mycotoxins in time. As many hybrids had very low or high to very high toxin contamination under the same environmental conditions, it is clear that, without knowing the resistance classification of the hybrid, its toxin behavior can hardly be forecasted. The forecasts shows higher toxin contamination in the northern hemisphere [35,37,42,84,85]. As the differences in hybrid resistance are very large in artificial inoculation tests [48,52,53] and also at natural infection and contamination verify this hypothesis, the combination of the two test regimes can lead to a decrease in toxin contamination. Therefore, the increasing resistance to toxigenic fungi can be an effective and excellent tool against the negative effects of warming climate. Under these conditions, hybrids with good resistance to heat, drought, and ear rot pathogens can be competitive and safer in middle Europe or further north, but also in regions where they cause severe problems now.

The correlations between infection, toxin contamination, and meteorological data show that, alone, warmer seasons do not interfere significantly with the production of toxin disease symptoms. For this, precipitation is also needed, and extra hot days also influence the results significantly, but differently on a monthly scale. The expression “global warming” simplifies the situation; therefore, it is better to avoid it. The forecast models use these data, so there is no problem in this respect.

### 4.6. Control Measurements

Artificial inoculation results clearly show resistance differences and the high deviations within season in natural toxin contamination support the view that higher resistance levels have a significant role in improving food and feed safety. Higher resistance levels can make fungicide control more efficient as it did in wheat [86,87]. The use of atoxic *A. flavus* isolates significantly reduces aflatoxin contamination [47,88]. We think that a higher resistance could help to further reduce aflatoxin contamination by a possible additive effect. This is a future research task. Higher resistance could help with the successful application of Bt maize hybrids. This could be supported by conservation agriculture [88,89], which could stabilize the resistance of maize to climatic stresses and indirectly reduce aflatoxin contamination. Similarly, it is supposed that plants with higher resistance to toxigenic fungi have a better tolerance to the higher disease pressure when cereals or maize were the previous crops, so the tillage without plugging could be realized with less toxin risks [90,91,92,93].

The harmful consequences of climate change can be significantly balanced both in regions where toxins are a daily problem now and in regions that are exposed to these threats in these years and later. For this reason, we need to apply different approaches combining them to have a better control, higher yield, and improved food and feed safety. The key is integrated plant management with increased resistance supported by a field-specific mix of the best possible optimizing of management practices for each field.Further, susceptible hybrids should be withdrawn from production and the variety registration should ban the registration and production of the susceptible hybrids.

## 5. Conclusions

Of grains harvested globally, about 10% (210 million tons) are lost to natural mycotoxin contamination [94]. This loss must be reduced. The first possibility to solve the toxin problem is that toxins be addressed immediately after harvest. Toxin contamination cannot be forecasted by visual ear rot data. In Hungary, in the last 8 years, DON, fumonisin, and aflatoxin contamination of preharvest origin have occurred regularly in freshly harvested grains of commercial maize hybrids. On the basis of this, different epidemics were identified. In this study, in 3 out of the 8 years, hybrids were found to have higher mean values of aflatoxin than the EU limits (20 μg/kg for feed). Differences in resistance could be a reason for the large differences in contamination among hybrids, but a significantly high toxin contamination can be a risk indicator. The breeder should also receive important feedback from the harvest toxin data. The combination of resistance with agronomy, and different plant protection means seems to be an important research task to improve food and feed safety. As the forecast of toxin contamination at a low-level infection level does not work, all samples should be controlled for toxins. This also has consequences for the breeding variety [95].

## Figures and Tables

**Table 1:** Mycotoxin contamination of moldy and regular corn samples in Hungary, 1993–1999 [24]. The line row for means and rate M/R was calculated by Mesterhazy (2022).

Year	Moldy Samples				Regular Samples			
	FUMO	DON	T-2	ZON	FUMO	DON	T-2	ZON
	μg/kg	μg/kg	μg/kg	μg/kg	μg/kg	μg/kg	μg/kg	μg/kg
1993	260	4330	200	1260	100	220	100	20
1994	6440	90	165	30	1520	50	50	8
1995	8650	2400	430	360	1600	370	180	100
1996	5520	3200	380	230	1380	420	160	150
1997	5940	3400	480	560	1170	470	220	190
1998	3960	n.d.	n.d.	n.d.	n.d.	n.d.	n.d.	n.d.
1999	n.d.	n.d.	n.d.	n.d.	1190	480	210	n.d.
Mean	5128	2684	331	488	1160	335	153	94
Rate M/R	4.42	8.01	2.16	5.21				

n.d. = Not detected.

**Table 3:** Correlation coefficients between responses of hybrids to different mycotoxins and ear rot data for *Fusarium* and *Aspergillus*, 2014–2020.

Years	ERF/ERA	ERF/DON	ERF/FUM	ERA/AFB1	DON/FUM	DON/AFB1	FUM/AFB1	n
2014	0.088	0.194	0.192	−0.125	0.523 *	−0.057	−0.146	20
2015	0.834 ***	−0.063	−0.180	−0.080	−0.197	−0.076	−0.146	23
2016	−0.092	0.119	0.146	−0.053	−0.098	−0.031	0.169	23
2017	0.016	−0.181	0.510 *	−0.207	−0.038	0.743 ***	0.037	23
2018	−0.094	−0.143	0.186	−0.067	−0.127	0.218	−0.172	23
2019	0.263	−0.240	0.301	−0.089	−0.034	−0.186	0.118	23
2020	0.480 *	−0.097	−0.230	−0.091	−0.092	−0.112	0.213	18
2021	0.0035	0.278	0.564 *	−0.277	0.066	−0.129	−0.158	14

*** *p* = 0.001, * *p* = 0.05, ERF = *Fusarium* natural visual ear rot; ERA = *Asp*. nat. visual ear rot; DON = deoxynivalenol; FUM = fumonisin B1 + B2; AFB1 = aflatoxin B1.

**Table 4:** Means of the hybrids (2014–2020) for natural infection and toxin production.

Year	Visual Ear Rot	Visual Ear Rot	Toxin		
	Fus%	Asp%	DON (mg/kg)	FUM B_1_ + B_2_ (mg/kg)	AFB1 (μg/kg)
2014	1.12	0.001	**6.29**	**20.79**	16
2015	1.21	0.040	0.14	**4.03**	**87**
2016	0.25	0.001	0.16	1.76	6
2017	0.47	0.040	0.25	1.82	**51**
2018	0.47	0.001	0.90	0.7	3
2019	0.18	0.001	**1.77**	1.16	4
2020	0.18	0.004	0.09	2.16	**316**
2021	0.09	0.000	0.71	0.87	0.86
Mean	0.50	0.011	1.29	4.16	60.48
Correlations		Fus. Ear rot %	Asp Ear rot %	DON (mg/kg)	FUM B_1_ + B_2_ (mg/kg)
Asp Ear rot %		0.4808			
DON mgt/kg		0.4812	−0.3351		
FUM B_1_ + B_2_ mg/kg		0.6731 *	−0.1161	0.9277 **	
AFB1 μg/kg		−0.0987	0.1103	−0.2897	−0.1047

** *p* = 0.01; * *p* = 0.05. A term in bold shows higher mean toxin than the EU limit, therefore signalizes an epidemic of that toxin.

**Table 6:** Symptom severity and natural toxin contamination of Valkür, 2017–2020.

Year	Fus.Visual Ear Rot	Asp.Visual Ear Rot	Toxins		
	%	%	DON (mg/kg)	FUM B_1_ + B_2_ (mg/kg)	AFB1 (μg/kg)
2017	0.02	0.000	0.22	0.81	0.010
2018	0.03	0.000	1.00	0.20	0.000
2019	0.07	0.000	2.40	0.00	0.000
2020	0.04	0.000	0.15	3.30	0.000
2021	0.03	0.000	0	1.48	0.001
Mean	0.04	0.000	0.76	1.16	0.0022

**Table 10:** Toxin data of the four control hybrids from natural contamination, 2017–2020.

Hybrid	DON (mg/kg)				Mean	Variance
	2017	2018	2019	2020		
Valkür	0.22	1.00	2.40	0.15	**0.94**	1.09
DKC 5830	0.00	0.00	4.35	0.00	**1.09**	4.73
Korimbos	0.60	4.20	6.80	0.17	**2.94**	9.88
DKC 4541	0.00	0.10	7.18	0.00	**1.82**	12.77
Mean	0.21	1.32	5.18	0.08	1.7	7.12
LSD 5%					ns	
Hybrid	FUM B_1_ + B_2_ (mg/kg)				Mean	Variance
	2017	2018	2019	2020		
Valkür	0.81	0.20	0.00	3.30	**1.08**	2.31
DKC 5830	0.18	0.00	2.84	3.28	**1.58**	2.98
Korimbos	0.83	0.30	1.39	0.22	**0.67**	0.29
DKC 4541	2.53	0.40	0.00	1.08	**1.00**	1.24
Mean	1.09	0.21	1.06	1.97	**1.08**	1.71
LSD 5%					ns	
Hybrid	AFB1 (μg/kg)				Mean	Variance
	2017	2018	2019	2020		
Valkür	10	0	0	0	**2.50**	25.00
DKC 5830	5	0	0	0	**1.25**	6.25
Korimbos	55	0	0	816	**217.75**	159,740.25
DKC 4541	17	0	0	0	**4.25**	72.25
Mean	21.75	0	0	204	**56.44**	39,960.93
LSD 5%					ns	

* **Bold**: the mean the data are marked.

## Data Availability

All data are available upon request from the corresponding author.

## Supplementary Materials

The following supporting information can be downloaded at: https://www.mdpi.com/article/10.3390/jof8101104/s1, Table S1. Visual and natural ear rot infection and toxin contamination of maize hybrids, 2014. Table S2. Visual and natural ear rot infection and toxin contamination of maize hybrids, 2015. Table S3. Visual and natural ear rot infection and toxin contamination of maize hybrids, 2016. Table S4. Visual and natural ear rot infection and toxin contamination of maize hybrids, 2017. Table S5. Visual and natural ear rot infection and toxin contamination of maize hybrids, 2018, ranking: DON. Table S6. Visual and natural ear rot infection and toxin contamination of maize hybrids, 2019. Table S7. Visual and natural ear rot infection and toxin contamination of maize hybrids, 2020, ranking: DON. Table S8. Visual and natural ear rot infection and toxin contamination of maize hybrids, 2021, ranking: DON.

## Abbreviations

AFB1	Aflatoxin B1
ANOVA	Analysis of variance
Asp	*Aspergillus*
Bt hybrid	Containing an insecticide gene from *B. thuringiensis*
DON	Deoxynivalenol
FER	*Fusarium* ear rot (mostly *F. verticillioides*)
FUM	Fumonisin
Fus	*Fusarium*
GER	Gibberella ear rot (mostly *F. graminearum*)
GM	Genetically modified
