# Supplementary material for: The Role of Preharvest Natural Infection and Toxin Contamination in Food and Feed Safety in Maize, South-East Hungary, 2014–2021
Source: J Fungi (Basel). 2022 Oct 19;8(10):1104. doi: 10.3390/jof8101104 (PMC9605659; doi:10.3390/jof8101104)
Supplement: Supplementary file 1 [file jof-08-01104-s001.zip › jof-1945856-supplementary.pdf]

Table S1. Visual and natural ear rot infection and toxin contamination of maize hybrids, 2014

Hybrid	Fus* visual ear rot	Asp visual ear rot	Toxin mg/kg			Toxin content for 1% ear rot, mg/kg	
	%	%	DON	FUM B1+B2	AFB1	DON%	FUM%
**LG3535**	0.46	0	0.47	1.16	0	1.02	2.52
Kamaria	1.97	0	0.72	5.07	0	0.37	2.57
LG3475	1.31	0	0.74	11.7	0	0.56	8.93
Siloking	2.9	0	0.74	27.55	0	0.26	9.50
PO412	0.52	0	0.85	5	0	1.63	9.62
P9915	0.67	0	1.02	13.18	0.006	1.52	19.67
DKC5276	1.37	0.03	1.08	42.15	0	0.79	30.77
DKC6031	0.46	0	1.25	12.29	0	2.72	26.72
LG30491	0.28	0	1.73	34.44	0.121	6.18	123.00
Korimbos	0.72	0	2.61	7.09	0	3.63	9.85
DKC5007	0.5	0	3.48	19.98	0.057	6.96	39.96
AXXYS	0.69	0	4.17	17.22	0.02	6.04	24.96
PR37MO1	0.87	0	6.11	17.01	0.021	7.02	19.55
DKC4717	1.61	0	8.9	14.25	0.025	5.53	8.85
PR37F73	1.81	0	9.26	21.45	0.007	5.12	11.85
PR38A24	0.84	0	11.55	25.93	0.003	13.75	30.87
Janett	1.12	0	13.24	35.91	0.008	11.82	32.06
P0216	1.85	0	14.53	26.81	0.044	7.85	14.49
P9528	0.96	0	15.86	45.78	0	16.52	47.69
P1114	1.47	0	27.47	31.86	0.002	18.69	21.67
Mean	1.12	0..001	6.29	20.79	0.016	5.90	24.75
LSD 5%	0.49	ns					

*Fus. = Fusarium, Asp = Aspergillus, n=20, data are ranked by DON content. EU limits for swine DON 0.9 mg/kg, FUM 5 mg/kg, aflatoxin 20 µg/kg, piglets: DON 0.9 mg/kg, FUM 5 mg/kg, AFB1 5 µg/kg). Green highlight: good for adult swine. **Bold names**: good for all toxins. All aflatoxin rates for a percent of ear infection were zero, the zero divisors were not reported for this rate.

Table S2. Visual and natural ear rot infection and toxin contamination of maize hybrids, 2015

Hybrid	Fus* visual ear rot	Asp visual ear rot	Toxin mg/kg			Toxin content for 1% ear rot, mg/kg		
	%	%	DON	FUM B_1+B_2	AFB1	DON	FUM	AFB1
**DKC4943**	0.79	0	0	0.29	0	0.00	0.37	**
**LG 30369**	0.99	0	0	0.77	0	0.00	0.78	
DKC 5276	0.87	0.03	0	5.49	0	0.00	6.31	0.00
DKC5031	1.65	0	0	11.13	0.06	0.00	6.75	
Toxxol Duo	1.82	0.08	0	5.39	0	0.00	2.96	0.00
**Janett**	0.28	0	0	0.51	0	0.00	1.82	
**DKC 4717**	0.54	0	0	2.07	0	0.00	3.83	
P0412	0.56	0	0	4.82	0.06	0.00	8.61	
**Konsens**	0.48	0.1	0	1.68	0	0.00	3.50	0.00
**Da Sonka**	1.02	0.01	0	1.63	0	0.00	1.60	0.00
P0023	1.11	0	0	6.64	0	0.00	5.98	
Oxxygen	1.09	0.11	0	7.11	0	0.00	6.52	0.00
DKC 4014	1.28	0.03	0	2.9	0.22	0.00	2.27	7.33
DKC4751	1.44	0	0	1.45	1.03	0.00	1.01	
Korimbos	1.47	0.33	0	0.81	0	0.00	0.55	0.00
DKC 5542	2.03	0	0	11.63	0.05	0.00	5.73	
P9549	3.1	0.1	0	11.42	0	0.00	3.68	0.00
**DKC 4590**	1.39	0	0.05	4.73	0	0.04	3.40	
**NK Octet**	1.61	0	0.08	3.35	0	0.05	2.08	
P9903	0.66	0.12	0.09	2.9	0.43	0.14	4.39	3.58
DKC5007	0.93	0	0.17	1.23	0	0.18	1.32	
P9911	1.7	0.03	0.23	3.65	0.14	0.14	2.15	4.67
Sy Affiniti	0.4	0	2.55	1.12	0	6.38	2.80	
Mean	1.17	0.04	0.14	4.03	0.087	0.30	3.41	1.56
LSD 5%	0.73	ns						

*Fus = Fusarium, Asp = Aspergillus, n= 23, data are ranked by DON content
Bold: lower than average for all toxins, green highlight: data below EU limits for adult swine. (Suggested EU feed limits for adults: DON 0.9 mg/kg, FUM 5 mg/kg, aflatoxin 20 µg/kg, piglets: DON 0.9 mg/kg, FUM µ mg/kg, AFB1 5 mg/kg), **Bold names**: good for all toxins. ** zero divisor

Table S3. Visual and natural ear rot infection and toxin contamination of maize hybrids, 2016

Hybrid	Fus* visual ear rot	Asp visual ear rot	Toxin mg/kg			Toxin content for 1% ear rot, mg/kg	
	%	%	DON	FUM B_1+B_2	$AFB1_1$	DON	FUM
**P 0725**	0.01	0	0	0.77	0.003	0.00	77.00
P 0412	0.05	0	0	5.29	0.007	0.00	105.80
**LG 30.389**	0.06	0	0	1.61	0.006	0.00	26.83
**Janett**	0.07	0	0	0.23	0	0.00	3.29
**DKC 5542**	0.08	0	0	1.06	0.001	0.00	13.25
**Korimbos**	0.11	0.09	0	2.03	0.001	0.00	18.45
P 9241	0.14	0	0	6.18	0.002	0.00	44.14
**Konsens**	0.19	0	0	2.28	0.001	0.00	12.00
**ES Sensor**	0.2	0	0	2.33	0	0.00	11.65
**P 1535**	0.2	0	0	1.51	0	0.00	7.55
**KWS 2376**	0.22	0	0	1.56	0	0.00	7.09
**Sy Ulises**	0.27	0	0	0.66	0.002	0.00	2.44
**DKC 4943**	0.27	0	0	1.06	0	0.00	3.93
**Cadixxio Duo**	0.31	0	0	1.08	0.01	0.00	3.48
**P 9537**	0.38	0	0	0.83	0	0.00	2.18
**DKC 4014**	0.5	0	0	0.53	0	0.00	1.06
**Futurixx**	0.18	0	0.12	0.19	0	0.67	1.06
**P 9903**	0.11	0	0.15	3.15	0	1.36	28.64
**DKC 4717**	0.25	0	0.4	0.82	0	1.60	3.28
**DKC 4541**	1.66	0	0.42	3.85	0	0.25	2.32
**DKC 6031**	0.06	0	0.57	2.23	0	9.50	37.17
**Toxxol Duó**	0.31	0	0.58	0.14	0.002	1.87	0.45
Sy Octavius	0.11	0	1.43	1.07	0.003	13.00	9.73
Mean	0.25	0.001	0.16	1.76	0.002	1.23	18.38
LSD 5%	0.26	ns					

*Fus = Fusarium, Asp = Aspergillus, n= 23, data are ranked by DON content. EU limits for swine DON 0.9 mg/kg, FUM 5 mg/kg, aflatoxin 20 µg/kg, piglets: DON 0.9 mg/kg, FUM 5 mg/kg, AFB1 5 µg/kg. **Bold names**: good for all toxins. Green highlight: good for adult swine

Table S4. Visual and natural ear rot infection and toxin contamination of maize hybrids, 2017

Hybrid	Fus*. visual ear rot	Asp visual ear rot	Toxin mg/kg			Toxin content for 1% ear rot, mg/kg		
	%	%	DON	FUM B_1+B_2	AFB1	DON	FUM	AFB1
Janett	0.25	0	0	1.12	0.114	0.00	4.48	**
**DKC 5830**	0.28	0	0	0.18	0.005	0.00	0.64	
**P 9241**	0.29	0	0	1.42	0.009	0.00	4.90	
Siló Star	0.3	0	0	0.53	0.077	0.00	1.77	
P 9537	0.36	0	0	1.85	0.022	0.00	5.14	
**RGT Prefixx**	0.38	0	0	1.46	0.006	0.00	3.84	
**DKC 4717**	0.42	0	0	1.24	0.014	0.00	2.95	
**DKC 4541**	0.42	0.07	0	2.53	0.017	0.00	6.02	0.24
DKC 5542	0.6	0	0	4.34	0.066	0.00	7.23	
Konsens	0.65	0	0	0.73	0.046	0.00	1.12	
**Fornad**	0.49	0.17	0	2.88	0.007	0.00	5.88	0.04
**P 9903**	0.7	0	0	0.83	0.005	0.00	1.19	
PR37F80	0.77	0	0	1.22	0.033	0.00	1.58	
P 9911	0.45	0.33	0	2.09	0.021	0.00	4.64	0.06
4517	0.82	0	0	10.21	0.058	0.00	12.45	
Szegedi 521	0.42	0	0.09	0.52	0.169	0.21	1.24	
DKC 4943	0.3	0.1	0.14	0.76	0.043	0.47	2.53	0.43
**Valkür**	0.02	0	0.22	0.81	0.01	11.00	40.50	
**DKC 4590**	0.65	0.1	0.37	2.22	0.002	0.57	3.42	0.02
**Cardixxio Duo**	0.44	0	0.39	0.36	0.004	0.89	0.82	
Korimbos	0.18	0	0.6	0.83	0.055	3.33	4.61	
P 0023	0.21	0.1	1.13	1.76	0.001	5.38	8.38	0.01
KK 4420	0.38	0	2.79	2.01	0.385	7.34	5.29	
Mean	0.45	0.04	0.25	1.82	0.051	1.27	5.68	0.13
LSD 5%	0.34	ns						

*Fus = Fusarium, Asp = Aspergillus, n= 23, data are ranked by DON content. EU limits for swine DON 0.9 mg/kg, FUM 5 mg/kg, aflatoxin 20 µg/kg, piglets: DON 0.9 mg/kg, FUM 5 mg/kg, AFB1 5 µg/kg. **Bold names**: good for all toxins. Green highlight: good for adult swine, **zero divisor

Table S5. Visual and natural ear rot infection and toxin contamination of maize hybrids, 2018, ranking: DON

Hybrid	Fus*. visual ear rot	Asp visual ear rot	Toxin mg/kg			Toxin content for 1% ear rot, mg/kg		
	%	%	DON	FUM B_1+B_2	AFB1	DON	FUM	AFB1
**Lg 30.452**	0.03	0	0	0.68	0	0.00	22.67	**
**Cardixxio Duo**	0.08	0	0	0	0.001	0.00	0.00	
**SY Zephir**	0.11	0	0	0.11	0.006	0.00	1.00	
**Armagnac**	0.11	0	0	0.52	0.001	0.00	4.73	
**DKC 5830**	0.13	0	0	0	0	0.00	0.00	
**P 9537**	0.14	0	0	1.24	0	0.00	8.86	
**DKC 5542**	0.14	0.02	0	1.14	0	0.00	8.14	0.00
**P 0412**	0.15	0	0	1.02	0	0.00	6.80	
P 9241	0.17	0	0	0	0.19	0.00	0.00	
**P 9911**	0.2	0	0	0.31	0	0.00	1.55	
**DKC 4590**	0.26	0	0	1.31	0.001	0.00	5.04	
**4517**	0.38	0	0	3.76	0	0.00	9.89	
**PR37F80**	0.39	0	0	0.55	0.005	0.00	1.41	
**Szegedi 521**	0.42	0	0	0.79	0.012	0.00	1.88	
**DKC 4717**	0.27	0	0.1	2.64	0.001	0.37	9.78	
**DKC 4541**	0.83	0	0.1	0.39	0	0.12	0.47	
**P 9903**	0.17	0	0.2	0	0	1.18	0.00	
**DKC 4943**	0.24	0	0.5	0.14	0	2.08	0.58	
Valkür	0.03	0	1	0.2	0	33.33	6.67	
Fornad	0.6	0	2.1	0.29	0.006	3.50	0.48	
Siló Star	0.11	0	2.6	0.11	0	23.64	1.00	
Korimbos	0.06	0	4.2	0.25	0	70.00	4.17	
Koregraf	0.12	0	9.9	0.64	0.07	82.50	5.33	
Mean	0.42	0.001.	0.9	0.7	0.013	9.42	4.37	0.00
LSD 5%	0.29							

*Fus. = Fusarium, Asp = Aspergillus, EU limits for swine DON 0.9 mg/kg, FUM 5 mg/kg, aflatoxin 20 µg/kg, piglets: DON 0.9 mg/kg, FUM 5 mg/kg, AFB1 5 µg/kg, **Bold names**: good for all toxins. ** zero divisor

Table S6. Visual and natural ear rot infection and toxin contamination of maize hybrids, 2019

Hybrid	Fus* visual ear rot	Asp visual ear rot	Toxin mg/kg			Toxin content for 1% ear rot, mg/kg		
	%	%	DON	FUM B_1+B_2	AFB1	DON	FUM	AFB1
**Koregraf**	0.09	0	0	0.36	0	**		0.00
**P0725**	0.16	0	0	0.63	0			0.00
**Kleopatras**	0.22	0	0	3.34	0			0.00
ES Lagoon	0.23	0	0	2.28	0.021			0.00
**Sy Zoan**	0.25	0	0	2.54	0			0.00
**DKC 4590 Waxy**	0.37	0	0	0.78	0			0.00
**Illango**	0.22	0	0.09	1.89	0	0.00	0.00	0.00
**ES Harmonium**	0.21	0	0.25	0	0.001	0.00	0.00	
**Szegedi 521**	0.21	0	0.25	0.1	0.006	0.00	0.02	0.00
**SY Zephir**	0.17	0	0.33	0	0	0.00	0.00	
Lexxtour Duo	0.2	0	0.52	1.47	0.065	0.00	0.13	0.00
**Kathedralis**	0.14	0	0.53	0.43	0	0.00	0.00	0.00
Sy Talisman	0.17	0	1.04	1.07	0.002	0.00	0.00	0.00
P9718E	0.06	0	1.13	0.24	0	0.00	0.00	0.00
P9415	0.12	0	1.16	1.35	0	0.00	0.00	0.00
Valkür	0.07	0	2.4	0	0	0.00	0.00	
Janett	0.24	0	2.93	3.73	0.002	0.00	0.00	0.00
Konfites	0.27	0.02	3.27	1.23	0	0.00	0.00	0.00
DKC5075	0.2	0.01	3.87	0.45	0	0.00	0.00	0.00
DKC 5830	0.16	0	4.35	2.84	0	0.00	0.00	0.00
Armagnac	0.15	0	4.65	0.61	0	0.00	0.00	0.00
Korimbos	0.05	0	6.8	1.39	0	0.00	0.00	0.00
DKC 4541	0.21	0	7.18	0	0	0.00	0.00	
Mean	0.18	0.001	1.77	1.16	0.004	0.00	0.01	0.00
LSD 5%	0.15							

*Fus. = Fusarium, Asp. = Aspergillus, n= 23, data are ranked by DON content. EU limits for swine DON 0.9 mg/kg, FUM 5 mg/kg, aflatoxin 20 µg/kg, piglets: DON 0.9 mg/kg, FUM 5 mg/kg, AFB1 5 µg/kg, **Bold names**: good for all toxins. ** zero divisor

Table S7. Visual and natural ear rot infection and toxin contamination of maize hybrids, 2020, ranking: DON

Hybrid	Fus*. visual ear rot	Asp visual ear rot	DON	FUM B_1+B_2	AFB1	Toxin content for 1% ear rot, mg/kg		
	%	%	mg/kg			DON	FUM	AFB1
**P9415**	0.35	0	0	0.26	0	0.00	**	0.00
**DKC 4541**	0.39	0.07	0	1.08	0	0.00	15.43	1.00
	0.29	0	0	1.9	0	0.00		2.00
**DKC5830**	0.17	0	0	3.28	0	0.00		3.00
**Sy Zoan**	0.11	0	0	2.17	0	0.00		4.00
**ES Harmonium**	0.29	0.017	0	0.59	0.006	0.00	34.71	5.00
**Sy Zephir**	0.15	0	0	0.59	0.006	0.00		6.00
**ES Lagoon**	0.15	0.01	0	0.88	0.018	0.00	88.00	7.00
**Armagnac**	0.12	0.01	0	0.88	0.018	0.00	88.00	8.00
Koregraf	0.1	0	0	10.9	0.703	0.00		9.00
P 0725	0.19	0	0	4	1.588	0.00		10.00
Illango	0.14	0.01	0	1.57	2.286	0.00	157.00	11.00
**Konfites**	0.14	0	0.12	3.22	0.004	0.86		12.00
**P9718E**	0.12	0.01	0.13	0	0.008	1.08	0.00	13.00
Valkür	0.04	0	0.15	3.3	0	3.75		14.00
Korimbos	0.1	0	0.17	0.22	0.816	1.70		15.00
**Sy Talisman**	0.36	0	0.4	2.68	0.002	1.11		16.00
Kleopatras	0.11	0	0.7	1.27	0.227	6.36		17.00
Mean	0.18	0.004	0.09	2.16	0.316	0.83	63.86	8.50
LSD 5%	0.15	ns						

*Fus. = Fusarium, Asp = Aspergillus, n= 18, data are ranked by DON content, EU limits for swine DON 0.9 mg/kg, FUM 5 mg/kg, aflatoxin 20 µg/kg, piglets: DON 0.9 mg/kg, FUM 5 mg/kg, AFB1 5 µg/kg, **Bold names**: good for all toxins. ** zero divisor

Table S8. Visual and natural ear rot infection and toxin contamination of maize hybrids, 2021, ranking: DON

Hybrid	Fus*. visual ear rot	Asp visual ear rot	DON	FUM B_1+B_2	AFB1	Toxin content for 1% ear rot, mg/kg		
	%	%	mg/kg	mg/kg	mg/kg	DON	FUM	AFB1
**P9415**	0.04	0.00	0.00	0.24	0.00	0.00	6.00	**
**DKC 4541**	0.12	0.00	0.00	0.81	0.00	0.00	6.75	
**SY Zephir**	0.05	0.00	0.00	0.08	0.00	0.00	1.60	
**DKC 4943**	0.07	0.00	0.00	0.25	0.00	0.00	3.57	
**KWS Inteligens**	0.05	0.00	0.00	0.67	0.00	0.00	13.40	
**P 0725**	0.16	0.00	0.00	0.40	0.00	0.00	2.50	
**Valkür**	0.03	0.00	0.00	1.48	0.00	0.00	49.33	
**P9978**	0.08	0.00	0.12	1.07	0.00	1.50	13.38	
**Cardixxio Duo**	0.08	0.00	0.14	0.58	0.00	1.75	7.25	
**Korimbos**	0.04	0.00	0.14	0.28	0.01	3.50	7.00	
**P 9911**	0.05	0.00	0.39	2.00	0.00	7.80	40.00	
**Fornad**	0.29	0.00	0.83	2.84	0.00	2.86	9.79	
Armagnac	0.11	0.00	3.50	0.65	0.00	31.82	5.91	
DKC 5542	0.13	0.00	4.77	0.83	0.00	36.69	6.38	
Mean	0.09	0	0.71	0.87	0.001	6.14	12.35	
LSD 5%	0.11	ns	0.13	0.08	0.0007			

*Fus. = Fusarium, Asp = Aspergillus, n= 18, data are ranked by DON content. EU limits for swine DON 0.9 mg/kg, FUM 5 mg/kg, aflatoxin 20 µg/kg, piglets: DON 0.9 mg/kg, FUM 5 mg/kg, AFB1 5 mg/kg, **Bold names**: good for all toxins. ** zero divisor
